# Prevalence of mandibular trauma leading to fracture in Eastern India: A retrospective study

**DOI:** 10.6026/973206300222102

**Published:** 2026-04-30

**Authors:** Rritam Ghosh, Kanad Chaudhuri, Debasree Boral, Debanti Giri, Sayan Chattopadhyay

**Affiliations:** 1 Department of Oral Medicine and Radiology, Dr. R. Ahmed Dental College and Hospital, Kolkata, West Bengal, India

**Keywords:** Age distribution, epidemiology, mandibular fractures, maxillofacial injuries, retrospective studies

## Abstract

Mandibular fractures pose a major public health burden because their prevalence, anatomical patterns and causes vary significantly across populations, complicating prevention and treatment planning. Therefore, it is of interest to analyze 1,500 radiographically and clinically confirmed mandibular fractures identified from 3,600 outpatient records (2019-2023) at R. Ahmed Dental College and Hospital, Kolkata, after excluding incomplete and pathological cases. Demographic data, etiology, fracture site distribution and fracture multiplicity were recorded and evaluated independently by two examiners using descriptive statistics and chi square tests at a 5% significance level. Young adult males, especially in the 21-30 year age group, were most frequently affected, with road traffic accidents as the leading cause and parasymphysis and condyle emerging as the predominant fracture sites, mostly as single site fractures. Thus, we document parasymphysis fractures in young men secondary to traffic accidents as the dominant pattern in this setting, underscoring the need for targeted road safety policies and maxillofacial trauma prevention strategies.

## Background:

The maxillofacial region is a highly vulnerable area to trauma because of its anatomical exposure and lack of protection, which often leads to functional and esthetic deficits [1]. These fractures have a substantial socio-economic cost in addition to causing morbidity [2]. Despite being the largest and strongest facial bone; the mandible is the most commonly fractured due to its exposed position, accounting for 36-59% of all maxillofacial fractures [3]. It fractures two to three times more frequently than other facial bones and the parasymphyseal, condylar and angle regions are common sites of fracture. RTAs, assaults, sports injuries and falls are etiological factors; RTAs are more common in developing nations, whereas assaults are more common in developed ones [4]. Variations persist across populations, despite numerous regional studies documenting patterns of mandibular fractures [5]. Therefore, it is of interest to examine the anatomical patterns, demographic distribution and etiology of mandibular fractures in our context.

## Materials and Methods:

## Study setting:

The retrospective study was conducted in the department of oral medicine and radiology at R. Ahmed Dental College and Hospital, Kolkata, after ethical approval from the Institutional Ethics Committee (IEC No. RADCH/EC/03/2023). We collected and organized data from the department of oral medicine and radiology's incident report book (IR Book) for 3,600 patients who presented to the hospital's outpatient department from various parts of west Bengal and nearby states over 4 years, from 2019 to 2023. The diagnosis of fracture was based on clinical history, signs and symptoms, visual findings, manual examination and interpretation of radiographs, as subsequently documented in the IR Book. Of these, 1,500 patients with clinically and radiographically confirmed mandibular fractures were identified and included in further analysis.

## Study design:

We used a retrospective observational design to examine the prevalence of mandibular fractures, their causes and their distribution. Because of this design, it wasn't necessary to perform a sample size calculation in advance, as all eligible case records from the study period were included. We looked at 1,500 patient records that met the eligibility criteria.

## Study groups and eligibility criteria:

We used a retrospective observational design to examine the prevalence of mandibular fractures, their causes and their distribution. Because of this design, it wasn't necessary to perform a sample size calculation in advance, as all eligible case records from the study period were included. We looked at 1,500 patient records that met the eligibility criteria. Some reasons for excluding people included incomplete medical records, pathological fractures and cases with insufficient radiographic documentation. Only fractures linked to trauma- related extractions were considered, while teeth removed due to periodontal disease or caries were not included.

## Methodology:

Some reasons for excluding people included incomplete medical records, pathological fractures and cases with insufficient radiographic documentation. Only fractures linked to trauma-related extractions were considered, while teeth removed due to periodontal disease or caries were not included.

## Outcome analysis:

The main results were a breakdown of mandibular fractures by cause, age, gender and location. Secondary outcomes examined links between the fracture site and its cause.

## Statistical analysis:

The data collected was first organized in Microsoft Excel 2021 and subsequently analyzed using IBM SPSS Statistics for Windows, version 26.0 (IBM Corp., Armonk, NY). Descriptive statistics, including frequencies and percentages, were employed to summarize categorical variables. Associations between categorical variables were assessed using the corrected Chi-square (χ^2^) test. A P-value ≤ 0.05 was considered statistically significant.

## Results:

First, the data was put into Microsoft Excel 2021. Then it was analyzed using IBM SPSS Statistics for Windows, version 26.0 (IBM Corp., Armonk, NY). To summarize categorical variables, descriptive statistics like frequencies and percentages were used. The corrected Chi- square (χ^2^) test was used to look at relationships between categorical variables. A P-value of less than or equal to 0.05 was thought to be statistically significant. Of 3600 cases screened, 1500 had mandibular fractures, representing 41.7% of cases. Of the 1500 people in this study, 755 (50.3%) were 30 years old or younger and 745 (49.7%) were older than 30. The average age of the participants was 33.39 years, with a standard deviation of 12.39 years. The median age was 30 years and the interquartile range (IQR) was 23.00 to 42.00 years. There was a significant age range within the study population, as evidenced by the minimum and maximum recorded ages of 14 and 70 years, respectively. With 1370 males (91.3%) and just 130 females (8.7%), the study sample showed a clear male predominance. The parapharyngeal region was the most commonly affected, accounting for 573 cases (26.7%) based on the fracture site distribution. Of them, 40 (1.9%) had bilateral paraymphysis and 533 (25.0%) had unilateral paraymphysis. 1486 cases (99.07%) had simple fractures, whereas 14 cases (0.93%) had comminuted fractures, according to the type of fracture sustained. When the number of fracture sites was analyzed, 582 people (38.8%) had multiple-site fractures, while 918 people (61.2%) had single-site fractures. With 1003 cases (67.0%) reported, RTA was the most frequent etiology. Subsequently, there were 307 cases (20.5%) of self-fall, 150 cases (10.0%) of physical assault, 27 cases (1.8%) of domestic violence, 10 cases (0.7%) of sports injuries and 1 case (0.1%) of miscellaneous causes (Table 1 - see PDF). The distribution of mandibular fracture sites by age and gender is contrasted in Figure 1. The most frequently affected areas in the ≤ 30-year age group were the unilateral angle (13.2%), the unilateral condyle (17.2%) and the unilateral paraymphysis (26.2%). Unilateral paranasal sinus (23.8%), condyle (19.6%) and angle (13.3%) fractures continued to be the most common among participants over 30. Males displayed a higher absolute frequency across all anatomical locations when the data were stratified by gender. The unilateral paraphysis (25%), unilateral condyle (18%) and angle (13%) were the most common locations in men. Even though they made up a smaller percentage of the cohort, female participants showed similar site distributions, with the most common sites being unilateral paraymphysis (25%), unilateral condyle (19%) and angle (12%). However, chi-square analysis showed that these differences in fracture-site distribution across age groups and genders were not statistically significant. A further analysis was conducted, shown in Table 2 (see PDF), to evaluate the relationship between the demographic variables (age group and gender) and the type of mandibular fracture (single-site vs. multiple-site). Among individuals aged ≤30 years, 463 (61.3%) had single-site fractures and 292 (38.7%) had multiple-site fractures. Similarly, among those aged >30 years, 455 (61.1%) had single-site fractures and 290 (38.9%) had multiple-site fractures. The difference between these two age groups was not statistically significant (P = 0.92). Regarding gender, 80 females (62%) had single-site fractures and 50 (38%) had multiple-site fractures. Among males, 838 (61.5%) had single-site fractures and 532 (38.8%) had multiple-site fractures. The association between gender and fracture type was also not statistically significant (P = 0.93).

## Discussion:

Of the 1,500 out of 3,600 maxillofacial trauma cases screened in this retrospective cohort study at a tertiary dental hospital in eastern India, 41.7% had mandibular fractures. This result is consistent with earlier findings that the mandible, due to its anatomical prominence and biomechanical weaknesses, is the most commonly fractured facial bone [2]. This trend has been consistently noted by Shankar *et al.* [2] Juncar *et al.* [7] Al Ahmed *et al.* [7] Adekeye *et al.* [8] and Holmes *et al.* [9]. The age distribution peaked among young adults (median age 30 years; 50.3% aged 30 years or younger). This observation is consistent with the enhanced mobility, a tendency toward risk-taking and overall exposure to vehicular and occupational hazards in this age group [2, 6 and 10]. The 91.3% male predominance from this cohort can be attributed to the well-documented gender bias in maxillofacial trauma, which has been attributed to greater outdoor activities, vehicular exposure and interpersonal risk among men in different regions [6, 10 and 11]. Although a reduced male-to-female gap has been reported in more developed settings [12, 13], most Indian and some other Asian cohorts still report a greater male preponderance, in keeping with the results from Shankar *et al.* [14] Shankar *et al.* [2] and Juncar *et al.* [6]. RTAs (67.0%) were the most common mechanism of injury, followed by falls (20.5%) and assaults (10.0%). All of these findings are in line with those of multicentre studies, highlighting the importance of poor road discipline and the high proportion of motorcyclists, as well as poor use of safety equipment [11]. Taiwanese data also show RTA predominance, with motorcycle crashes a key contributor, underscoring transport-mix effects on epidemiology [5]. In contrast, European cohorts have documented interpersonal violence as the dominant mechanism, illustrating region-specific social and policy determinants [6, 15]. Cirignano *et al.* have reported a higher frequency of trauma due to interpersonal conflict, further illustrating geographic variability in etiological patterns [16]. The distribution of sites within our cohort was predominantly observed in the parasymphysis (26.9%) and condyle (23.3%), followed by the angle (13.2%) and body (12.1%). Smaller contributions were noted from fractures of the symphysis, dentoalveolar, ramus and coronoid. This distribution pattern aligns with various studies from India and Taiwan, which also identify the parasymphysis and condyle as the most frequently affected sites [5, 10 and 11]. Biomechanically, areas of tensile stress concentration at the curvature of the mandible and the region of the canine root are predisposed to failures at the parasymphyseal area. In contrast, high-velocity impacts tend to direct forces towards the condylar neck, which accounts for the common occurrence of pairs involving the parasymphysis and the condyle [11, 17]. International diversity continues to exist, characterized by angle-dominant patterns in certain Western series and body-dominant patterns in others. RTAs were the leading cause in our study (67.0%), followed by falls (20.5%). This may reflect differences in impact mechanisms, helmet use and local transportation patterns [18]. The fractures in our cohort were mostly simple (99.1%) and an overwhelming majority of patients had single-site involvement (61.2%). These statistics are generally consistent with data from district-level hospitals in India and various reports from Asia. However, certain specialized centers that handle high-energy polytrauma cases report a greater prevalence of multiple-site injuries [2, 19]. No notable differences were noted in single versus multiple fracture presentation by age group or sex in our data, reinforcing the notion that the multiplicity of fractures may be influenced more by the direction and energy of the impact rather than solely by demographic factors [11]. When categorized by age and gender, the relative frequencies of parasymphysis, condyle and angle fractures were consistent across strata. Furthermore, the absence of statistical significance regarding distributional differences is consistent with findings in other studies, in which the mechanism and direction of force, rather than demographic factors alone, account for site preferences [11]. Our assault subgroup (10.0%) exhibited a greater proportion of angle involvement, aligning with existing literature that associates low-velocity blunt interpersonal violence with angle fractures. In contrast, high-velocity RTA mechanisms tend to favor condylar and symphyseal/parasymphyseal locations [5, 20]. Comparative prevalence statistics underscore the role of regional transport and policy environments: our RTA share (67.0%) is close to that of Kanala *et al.* from India (70%) and the Northern Taiwan series, which emphasized motorcycle frequency. European cohorts tend to document assault predominance, as well as a growing risk of alcohol abuse [5, 6, 10 and 15]. These contrasts underline the importance of regional adaptation of injury-prevention strategies. Our results also echo smaller Indian hospital cohorts in which parasymphysis is first, with condylar associations, a pattern attributed to force transmission along the arch, with tensile failure at the neck of the condyle [11, 21]. Studies emphasizing angle or body predominance may be due to differences in helmet types (i.e., half-helmets leaving the anterior mandible exposed), impact vector, or case-mix in referral hospitals [10]. From a system standpoint, the RTA burden justifies multi-modal public-health interventions: enforcement of helmet/seat-belt laws, targeted alcohol-impaired-co-driver countermeasures and infrastructure changes, all of which have been used successfully to reduce maxillofacial trauma in various locales [10, 15]. For the clinician, the high occurrence of parasymphysis-condyle patterns highlights the importance of careful occlusal and temporomandibular joint evaluation and a sensitive imaging protocol for concomitant condylar injury even when the primary impact is anterior [19]. Studying the limitations of this study, we recognize that it was retrospective, single-center and dependent on records, which may not have adequately documented important confounders, such as the use of protective devices, the level of intoxication and precise collision details. However, the substantial sample size and the consistency of patient care and documented information within the institutional departmental registry provided sufficient internal reliability for our pattern analysis. Study designs that prospectively collect information on protective device use, blood alcohol concentration and crash details would provide more reliable data to help us better identify modifiable risk factors in this group of patients.

## Conclusion:

We show a high incidence of mandibular fractures among young adult males due to RTAs. The parasymphysis and condyle are the commonly involved parts. The majority of cases were uncomplicated, localized injuries. Thus, we show the importance of road safety, targeted preventive education and comprehensive clinical evaluation to prevent current biomechanical fracture patterns.
